# Evidence on anti-malarial and diagnostic markets in Cambodia to guide malaria elimination strategies and policies

**DOI:** 10.1186/s12936-017-1807-y

**Published:** 2017-04-25

**Authors:** ACTwatch Group, Louis Akulayi, Angela Alum, Andrew Andrada, Julie Archer, Ekundayo D. Arogundade, Erick Auko, Abdul R. Badru, Katie Bates, Paul Bouanchaud, Meghan Bruce, Katia Bruxvoort, Peter Buyungo, Angela Camilleri, Emily D. Carter, Steven Chapman, Nikki Charman, Desmond Chavasse, Robyn Cyr, Kevin Duff, Gylsain Guedegbe, Keith Esch, Illah Evance, Anna Fulton, Hellen Gataaka, Tarryn Haslam, Emily Harris, Christine Hong, Catharine Hurley, Whitney Isenhower, Enid Kaabunga, Baraka D Kaaya, Esther Kabui, Beth Kangwana, Lason Kapata, Henry Kaula, Gloria Kigo, Irene Kyomuhangi, Aliza Lailari, Sandra LeFevre, Megan Littrell, Greta Martin, Daniel Michael, Erik Monroe, Godefroid Mpanya, Felton Mpasela, Felix Mulama, Anne Musuva, Julius Ngigi, Edward Ngoma, Marjorie Norman, Bernard Nyauchi, Kathryn A. O’Connell, Carolyne Ochieng, Edna Ogada, Linda Ongwenyi, Ricki Orford, Saysana Phanalasy, Stephen Poyer, Justin Rahariniaina, Jacky Raharinjatovo, Lanto Razafindralambo, Solofo Razakamiadana, Christina Riley, John Rodgers, Andria Rusk, Tanya Shewchuk, Simon Sensalire, Julianna Smith, Phok Sochea, Tsione Solomon, Raymond Sudoi, Martine Esther Tassiba, Katherine Thanel, Rachel Thompson, Mitsuru Toda, Chinazo Ujuju, Marie-Alix Valensi, Vamsi Vasireddy, Cynthia B. Whitman, Cyprien Zinsou, Sochea Phok, Dysoley Lek

**Affiliations:** 1 Population Services International, 1120 19th St NW, Suite 600, Washington, DC 20036 USA; 2 PSI Cambodia (PSK), No. 29, Street 334, Boeung Keng Kang I, Chamcar Mon, Phnom Penh, Kingdom of Cambodia; 3 The National Centre for Parasitology, Entomology and Malaria Control, 477 Betong Street (Corner St. 92), Village Trapangsvay, Sanakat Phnom Penh Thmey, Khan Sen Sok, Phnom Penh, Kingdom of Cambodia; 4 School of Public Health, National Institute of Public Health, Phnom Penh, Kingdom of Cambodia

**Keywords:** Malaria elimination, Case management, Anti-malarial, ACT, Private sector, Cambodia

## Abstract

**Background:**

Understanding Cambodia’s anti-malarial and diagnostic landscape in 2015 is critical for informing and monitoring strategies and policies as Cambodia moves forward with national efforts to eliminate malaria. The aim of this paper is to present timely and key findings on the public and private sector anti-malarial and diagnostic landscape in Cambodia. This evidence can serve as a baseline benchmark for guiding implementation of national strategies as well as other regional initiatives to address malaria elimination activities.

**Methods:**

From August 17th to October 1st, 2015, a cross sectional, nationally-representative malaria outlet survey was conducted in Cambodia. A census of all public and private outlets with potential to distribute malaria testing and/or treatment was conducted among 180 communes. An audit was completed for all anti-malarials, malaria rapid diagnostic tests (RDT) and microscopy.

**Results:**

A total of 26,664 outlets were screened, and 1303 outlets were eligible and interviewed. Among all screened outlets in the public sector, 75.9% of public health facilities and 67.7% of community health workers stocked both malaria diagnostic testing and a first-line artemisinin-based combination therapy (ACT). Among anti-malarial-stocking private sector outlets, 64.7% had malaria blood testing available, and 70.9% were stocking a first-line ACT. Market share data illustrate that most of the anti-malarials were sold or distributed through the private sector (58.4%), including itinerant drug vendors (23.4%). First-line ACT accounted for the majority of the market share across the public and private sectors (90.3%). Among private sector outlets stocking any anti-malarial, the proportion of outlets with a first-line ACT or RDT was higher among outlets that had reportedly received one or more forms of ‘support’ (e.g. reportedly received training in the previous year on malaria diagnosis [RDT and/or microscopy] and/or the national treatment guidelines for malaria) compared to outlets that did not report receiving any support (ACT: 82.1 and 60.6%, respectively; RDT: 78.2 and 64.0%, respectively).

**Conclusion:**

The results point to high availability and distribution of first-line ACT and widespread availability of malaria diagnosis, especially in the public sector. This suggests that there is a strong foundation for achieving elimination goals in Cambodia. However, key gaps in terms of availability of malaria commodities for case management must be addressed, particularly in the private sector where most people seek treatment. Continued engagement with the private sector will be important to ensure accelerated progress towards malaria elimination.

**Electronic supplementary material:**

The online version of this article (doi:10.1186/s12936-017-1807-y) contains supplementary material, which is available to authorized users.

## Background

Over the past decade, malaria interventions have made substantial progress in Cambodia, demonstrated by rapid declines in the malaria burden since the early 2000s, with reported cases decreasing by approximately 50% between 2004 and 2014 [1]. However, of Cambodia’s 25 provinces, 21 are still considered to be endemic, and an estimated 48% of the population live in high transmission areas [2]. Furthermore, there has been a resurgence of malaria cases between 2014 and 2015, from 44,748 to 56,371, and resistance to artemisinin-based combination therapy (ACT) continues to threaten progress towards national malaria strategies [3]. This situation is all the more pressing given the country’s recent commitment to eliminate malaria by 2020, as coverage of appropriate case management in the context of malaria elimination strategies will be critical to achieving this goal [1].

In 2016, Cambodia’s National Centre for Parasitology, Entomology and Malaria Control (CNM) released the Malaria Elimination Action Framework, 2016–2020, (MEAF) outlining the country’s strategies and plans to achieve elimination of *Plasmodium falciparum* and multi-drug resistant malaria by 2020 [1]. Several key objectives are described in the MEAF, including 100% parasitological diagnosis for all suspected cases and effective, efficacious treatment of all confirmed uncomplicated malaria cases using first-line ACT: dihydroartemisinin-piperaquine (DHA PPQ) or artesunate–mefloquine (ASMQ) fixed-dose combination (FDC). For *P. falciparum* infections or mixed infections that include *P. falciparum*, the MEAF stipulates use of a single low-dose of primaquine. For *P. vivax* infections, a standard dose of primaquine for up to 14 days is indicated in order to prevent relapse. The MEAF stipulates that glucose-6-phosphate dehydrogenase (G6PD) testing should be conducted prior to administering primaquine treatment for *Plasmodium vivax* cases.

The MEAF also outlines several key strategies to ensure readiness of public and private facilities to adhere to the national treatment guidelines for malaria [1]. Malaria treatment or referral services will be available at all public health facilities, licensed private sector providers, trained village malaria workers (VMW), mobile malaria workers, and military medical services. In the public sector, the VMW programme, which provides malaria diagnosis and treatment among remote communities through a community-based health workforce, will be scaled up. The number of villages with at least one VMW will almost double, from 2539 currently to 4528 over the course of the next 5 years. In addition, these community-based providers will be allowed to administer low dose primaquine for *P. falciparum* to reduce malaria transmission and for radical cure of *P. vivax.* The VMW will also receive training on malaria, case management, counseling, and health education.

In the private sector, where most patients in Cambodia seek treatment [4], the strategy outlines the scale-up of the existing public–private mix (PPM) programme by mapping all existing providers and registering new providers. Refresher courses on early malaria diagnosis, treatment, referral, and reporting will be conducted every 2 years for PPM programme providers. The strategy also specifies that providers that do not qualify for the PPM programme will not be allowed to provide or sell anti-malarials or diagnostics. However, efforts will also be made to identify and select unlicensed private providers that can be targeted for licensing so that they can be registered under the PPM programme. Several initiatives will be implemented to enforce the regulation of private sector service providers through the Department of Drug and Food (DEF) and the Anti-Economic Crime Police. Finally, the strategy also stipulates that there will be increased efforts to ban the import and sale of anti-malarial drugs that are not in the national malaria treatment guidelines [1]. This will be implemented by keeping the DDF updated on the anti-malarial drugs that are not included in the national malaria treatment guidelines.

These current national strategies outlined in the MEAF build on the country’s earlier efforts to promote expansion of the public sector and increased regulation of the private sector [5, 6]. In the public sector, the VMW programme has been a key strategy for increasing access to malaria commodities among remote rural populations. By 2014, a decade after it was piloted in 300 villages, the VMW programme covered over 1600 villages and 130 mobile communities across 17 malaria endemic provinces [1]. Private sector engagement has been in place since 2002, through national distribution of subsidized anti-malarials and rapid diagnostic testing (RDT) for private sector providers across the country [6]. In 2008, oral artemisinin monotherapy was banned, with several hard-line strategies in place to enforce the policy [7]. Increased regulation of the private sector commenced around this time, to reduce the role of unauthorized private sector providers, including drug stores and general retailers, in anti-malarial distribution, while continuing to support distribution by registered private for-profit health facilities and pharmacies. In 2011, the CNM and the Ministry of Health (MoH) established the aforementioned PPM programme to further engage the private sector and provided subsidized commodities, training, and supervision. The PPM programme was subsequently scaled up in 2014, with nearly 1200 licensed private providers enrolled across 34 operational districts (ODs) out of a total of 45 malaria endemic ODs [1]. Indeed, efforts to date by the CNM and other implementing partners have been highly successful in strengthening and shaping Cambodia’s anti-malarial and malaria diagnostic landscape. Supply-side evidence from 2013 has shown an increase in the widespread distribution of first-line ACT, successful removal of oral artemisinin monotherapy, a decrease in the number of unregulated outlets stocking anti-malarials, and an increase in public sector anti-malarial market share and composition, namely through the VMW programme [5].

Evidence on the role and performance of the public and private sectors will provide a baseline benchmark for guiding implementation of national strategies. The ACTwatch project, implemented since 2008 across a number of countries by Population Services International (PSI), provides timely, relevant, and high quality anti-malarial market evidence to inform and monitor national and global policy, strategy, and funding decisions for improving malaria case management [8, 9]. In 2015, an ACTwatch survey was implemented in Cambodia. The evidence generated from this project provides an opportunity to present contemporary market intelligence data on Cambodia’s anti-malarial and diagnostic landscape as a means to inform and monitor strategies and policies as the country moves forward with elimination activities. The evidence from this survey can also help to frame the anti-malarial and diagnostic market in the context of the MEAF strategies, as well as other regional and country-specific initiatives to accelerate progress towards elimination of malaria. The objectives of this paper are twofold: (1) to describe contemporary public and private sector readiness (availability of malaria commodities) and performance (market share) for malaria case management; and (2) to compare private sector readiness and provider knowledge between outlets that reportedly received supportive interventions (e.g. access to malaria commodities, or training) with private outlets that did not receive these interventions.

## Methods

### Design and sampling

The study population was defined as all outlets with the ‘potential’ to sell or distribute anti-malarial medicines and/or provide malaria blood testing. The methodology of ACTwatch adopts a more inclusive, rather than exclusive, way of determining outlet types for the study, by including a broad list of outlet types that may or may not sell or distribute anti-malarial medicines and/or provide malaria blood testing. While some outlets are expected to have anti-malarials, other outlets, such as general retailers, may be theorized to not stock these medicines. Such outlets are, however, included in the sample as a means to investigate this theory and to determine if these outlets do indeed contribute to malaria case management in a given country. Outlet types that are included in the sample are determined according to each specific country context. The outlet types that were included in the Cambodia survey are described in Table 1. Outlets that did not serve the general public (e.g. military facilities) were excluded from the outlet survey, but military and police facilities that also served the general public were included.

**Table 1:** Outlet types and definitions

Sector and facility type	Definition
Public sector	
Public health facilities	Referral hospitals, health centers, former district hospitals, health posts, NGO/mission/faith-based hospitals, NGO/mission/faith-based clinics, and NGO/mission/faith-based diagnostic laboratories
Community health workers	Community-based volunteers, either Village Malaria Workers, Mobile Malaria Workers, or Plantation Malaria Workers, who are equipped with anti-malarial treatment and malaria blood testing
Private sector	
Private for-profit health facilities	Private hospitals, clinics, polyclinics, cabinets, health care rooms, and private diagnostic laboratories and would be expected to have been registered in country
Pharmacies	Regulated by a national regulatory authority and staffed by pharmacists or qualified health practitioners. These include pharmacies, clinical pharmacies, depot A, and depot B. These may or may not be licensed by a national regulatory authority
Drug stores	Drug stalls in rural markets or shops that primarily sell medicines. These outlets are not guaranteed to be staffed by qualified health dispensers or practitioners and are not typically licensed by a national regulatory authority
General retailers	Grocery stores and village shops and are not licensed by a national regulatory authority
Itinerant drug vendors	Mobile providers found primarily in rural areas, typically working within a radius of their home. They are not registered with any national regulatory authority. Some itinerant drug vendors operate with both a fixed location and a mobile service, while others operate solely through a mobile service.

In 2014, the World Health Organization (WHO) used available evidence about artemisinin resistance to define a 3-tier stratification system for targeting action to address drug resistance [10]. This tier system replaced the previous zone stratification used in Cambodia [11]. Areas designated as Tier 1 were prioritized for immediate multifaceted response to contain or eliminate resistance. Areas designated as Tier 2 were prioritized for intensified malaria control to reduce transmission and/or limit the risk of emergence or spread of resistant parasites. Tier 3 areas had no evidence of artemisinin resistance and limited contact with Tier 1 areas. Malaria control in these areas focused on vector control, increasing coverage with confirmatory testing and treatment with quality-assured ACT [12]. The Cambodia outlet survey was stratified to deliver estimates for domains according to this tier stratification system, with the first research domain designated as tier 1 provinces and the second research domain designated as tier 2 provinces.

From a list of all communes in each research domain, the required number of units was selected with probability proportional to size (PPS). Selection of units with PPS was completed based on population estimates obtained from a 2010 Ministry of Planning projection based on findings from the 2008 national census. The sampling frames for each tier excluded communes that were located in non-malaria-endemic areas according to information provided by the CNM.

Within each commune, a census of all outlets with the potential to sell or distribute anti-malarials and/or provide malaria blood testing was conducted. Outlets were eligible for a provider interview and malaria product audit if they met at least one of three study criteria: (1) one or more anti-malarials reportedly in stock on the day of the survey; (2) one or more anti-malarials reportedly in stock within the three months preceding the survey; and/or (3) malaria RDT in stock or malaria microscopy available on the day of the survey.

### Sample size

Sample size was determined to estimate with precision (±10% points) two key indicators among public and private outlets: (1) proportion of outlets with first-line anti-malarial treatments available, among outlets with anti-malarial(s) in stock on the day of the survey; and (2) proportion of outlets with malaria blood testing (RDT or microscopy) available, among outlets with anti-malarial(s) in stock on the day of the survey or within the past 3 months. Estimates from the 2013 ACTwatch outlet survey were used to complete these calculations. A sample size of 80 Tier 1 and 80 Tier 2 communes was anticipated to yield the minimum required numbers of outlets.

### Data collection

Standard procedures used by the ACTwatch project to implement surveys have been described elsewhere [27]. Interviewers, supervisors, and quality controllers received training that included an orientation to the study and questionnaire, classroom training on completing anti-malarial and RDT audits, and a field exercise. Following training, data collection was implemented from August 17th to October 1st, 2015. The outlet survey was conducted using handheld devices with an Android operating system and electronic forms created using DroidDB (SYWARE, Inc., Cambridge, MA, USA). The questionnaire was translated from English to Khmer, and translated back into English to resolve any discrepancies prior to the survey implementation.

A series of screening questions were administered at all outlets to determine eligibility for the survey. Outlets where anti-malarial medicines were reportedly sold and/or malaria blood testing was reportedly available were invited to participate in the survey. Following informed consent procedures, an audit of all available anti-malarial medicines and RDT was conducted. In addition to the product audit, a series of questions was administered to the senior-most provider regarding malaria case management knowledge and practices as well as provider training and qualifications and reporting on malaria caseload data. Up to three visits were made to all outlets to complete the screening process, audit, and provider interview as needed.

### Data analysis

Electronic data were imported to a master dataset using Microsoft Access (Microsoft Corporation, Redmond, Washington, USA), and records were triangulated with questionnaires, field supervisor tracking records, and daily activity logs completed by interviewers. All data cleaning and analysis was completed using Stata 12.1 (StataCorp, College Station, TX, USA). Sampling weights were applied to account for variations in probability of selection, and standard error estimation accounted for clustering at the commune level.

Indicators were produced according to ACTwatch standards that have been implemented across time and country studies and which have been described in detail elsewhere [8, 9]. Availability of any anti-malarial was calculated with a denominator of all screened outlets. In the public sector, the availability of specific types of anti-malarials was calculated using the denominator of all screened outlets, given that anti-malarials should be available at all public health facilities and community health workers (CHW) designated as VMW. Availability of specific anti-malarial categories in the private sector was calculated using a denominator of private sector outlets stocking any anti-malarial.

The volumes of each anti-malarial that were reportedly distributed in the week prior to the survey, according to provider reports, were standardized into adult equivalent treatment doses (AETD) to allow for comparisons between medicines, based on WHO treatment guidelines for the amount of active ingredient required to treat an adult weighing 60 kg [13]. These standardized AETD volumes were then used to calculate market share for each anti-malarial category. Audited medicines that were missing information required to calculate AETD (strength or amount distributed) were excluded from this indicator.

Private sector outlet support status (the presence or absence of support) was calculated according to multiple provider self-reported variables. An outlet was considered as having received any support if the provider reported one or more of the following: (1) outlet received subsidized anti-malarials and/or malaria RDT; (2) at least one provider at the outlet received training on the national treatment guidelines for malaria or malaria diagnosis within the previous year; (3) outlet received a supervisory/regulatory visit within the previous year; and/or (4) outlet reports malaria caseload data to the government or a non-governmental organization. Support status was very likely to be correlated with outlet type, given that governmental and non-governmental organizations target only registered private health facilities and pharmacies for support and do not typically support unauthorized drug stores, general retail outlets, or itinerant drug vendors. Availability and provider knowledge indicators for supported and non-supported outlets were therefore adjusted for outlet type using logistic regression to produce adjusted predicted probabilities. Anti-malarial availability indicators were calculated among outlets with any anti-malarial in stock on the day of the survey. Malaria blood testing availability and provider knowledge of first-line treatment indicators were calculated among outlets with any anti-malarial in stock on the day of the survey or within the three months prior to the survey.

## Results

### Sample description

A total of 26,664 outlets were screened to assess eligibility for the outlet survey, and only 51 outlets refused screening or survey participation. Of the outlets screened, 604 (2.3%) were in the public sector, and 26,060 (97.7%) were in the private sector. A total of 1303 outlets were eligible and interviewed, and only five outlets met eligibility criteria but did not complete the interview. Of the eligible outlets, 557 (42.7%) were in the public sector, and 746 (57.3%) were in the private sector.

Of the 1303 outlets interviewed, 858 (65.8%) were stocking at least one anti-malarial on the day of the survey, 1112 (85.3%) were stocking at least one anti-malarial either on the day of the survey or within the previous three months, and 191 (14.7%) were stocking a malaria diagnostic test (either RDT or microscopy) but did not stock anti-malarial medicines on the day of the survey or within the previous three months.

In total, 164 outlets reported distributing an anti-malarial during the week prior to the survey, and 427 outlets reported providing or distributing a malaria diagnostic test in the week prior to the survey. Table 2 shows a detailed breakdown of the screening, eligibility, and interview results across outlet types and sectors. Provider characteristics are included in Additional file 1.

**Table 2:** Outlet survey sample

	Public health facility	Community health workers	Private not-for-profit facility	All public sector	Private for-profit health facility	Pharmacy	Drug store	General retailer	Itinerant drug vendor	All private sector	All outlets
Number of outlets											
Screened	173	430	1	604	668	290	338	23,840	924	26,060	26,664
Eligible and interviewed	142	415	0	557	327	99	46	39	235	746	1303
Refused	1	0	0	1	2	1	1	43	3	50	51
Eligible but not interviewed (interview non-participation)	0	1	0	1	1	2	0	1	0	4	5
Number of interviewed outlets											
With at least one anti-malarial	137	330	n/a	467	186	45	22	29	109	391	858
With at least one anti-malarial, or at least one anti-malarial in the past 3 months	140	402	n/a	542	237	74	34	39	186	570	1112
With malaria blood testing, but no anti-malarials in stock on the day of the survey or in the previous 3 months	2	13	n/a	15	90	25	12	0	49	176	191

### Availability in the public sector

Table 3 shows a detailed breakdown of malaria blood testing availability, first-line anti-malarial availability, and readiness for malaria case management across screened public sector outlets. Availability of malaria diagnostics was relatively high in the public sector, with 85.9% of all public sector outlets stocking either malaria RDT or microscopy, and this was highest among CHW (87.2%). Availability of malaria RDT was higher than microscopy, with 85.8% of public sector outlets stocking malaria RDT and only 7.2% reporting availability of malaria microscopy. Malaria microscopy was available in only 27.8% of public health facilities.

**Table 3:** Availability of malaria commodities and readiness for case management in the public sector, among all screened outlets

	Public health facility	Community health workers	All public sector
	N = 173	N = 430	N = 603
	% (95% CI)	% (95% CI)	% (95% CI)
Diagnostics			
Any malaria blood testing	82.3 (74.9, 87.8)	87.2 (82.7, 90.6)	85.9 (82.1, 89.0)
Malaria microscopy	27.8 (21.0, 35.8)	0.3 (0.1, 1.8)	7.2 (5.3, 9.7)
RDT	81.7 (74.3, 87.4)	87.2 (82.7, 90.6)	85.8 (81.9, 88.9)
Anti-malarials			
Any first-line ACT (DHA PPQ and/or ASMQ FDC)	76.5 (67.8, 83.5)	74.1 (66.2, 80.7)	74.7 (68.3, 80.1)
DHA PPQ	76.5 (67.8, 83.5)	74.1 (66.2, 80.7)	74.7 (68.3, 80.1)
ASMQ FDC	0.0 (–)	0.0 (–)	0.0 (–)
Primaquine	0.0 (–)	0.0 (–)	0.0 (–)
Other non-artemisinin therapy	8.9 (4.9, 15.5)	0.0 (–)	2.2 (1.3, 3.9)
IV/IM artesunate	0.0 (–)	n/a	0.0 (–)
Readiness			
Availability of first-line ACT and malaria blood testing	75.9 (67.1, 82.9)	67.7 (60.2, 74.3)	69.7 (63.6, 75.1)
Availability of first-line ACT, blood testing not available	0.7 (0.2, 2.4)	6.4 (4.1, 9.9)	5.0 (3.2, 7.6)

Availability of first-line ACT in the public sector was slightly lower than diagnostic availability, with just under three-fourths of all public sector outlets (74.7%) stocking any first-line ACT on the day of the survey. Among these outlets, 100% of the first-line ACT audited was DHA PPQ, as no outlets were stocking ASMQ FDC. Primaquine was not available in any public sector outlets.

Three-fourths of public health facilities (75.9%) had both malaria diagnostic testing and a first-line ACT. Malaria case management readiness was lower among CHW, with only 67.7% of CHW stocking both malaria testing and first-line treatment.

### Availability in the private sector

Table 4 shows a detailed breakdown of availability of any anti-malarial among all private sector outlets, followed by availability of malaria blood testing and anti-malarials among anti-malarial-stocking private sector outlets.

**Table 4:** Availability of malaria commodities in the private sector

	Private for-profit health facility	Pharmacy	Drug store	General retailer	Itinerant drug vendor	All private sector
	% (95% CI)	% (95% CI)	% (95% CI)	% (95% CI)	% (95% CI)	% (95% CI)
Among all screened outlets, availability of	**N = 668**	**N = 290**	**N = 338**	**N = 23,840**	**N = 924**	**N = 26,060**
Any anti-malarial	31.0 (26.1, 36.3)	20.5 (14.8, 27.6)	6.6 (4.2, 10.1)	0.2 (0.1, 0.4)	15.1 (11.4, 19.8)	1.8 (1.5, 2.1)
Among anti-malarial-stocking outlets or outlets stocking anti-malarials in the past 3 months, availability of	**N = 237**	**N = 74**	**N = 34**	**N = 39**	**N = 186**	**N = 570**
Diagnostics						
Any malaria blood testing	83.0 (76.8, 87.8)	70.1 (58.2, 79.7)	60.9 (40.8, 77.9)	0.0 (–)	60.4 (50.6, 69.4)	64.7 (58.5, 70.3)
Malaria microscopy	15.6 (10.9, 22.0)	4.8 (1.6, 13.1)	4.0 (0.6, 21.7)	0.0 (–)	5.6 (3.2, 9.8)	8.7 (6.4, 11.9)
RDT	81.3 (74.8, 86.5)	70.1 (58.2, 79.7)	60.9 (40.8, 77.9)	0.0 (–)	59.8 (50.0, 68.9)	63.8 (57.6, 69.6)
Among anti-malarial stocking outlets, availability of	**N = 186**	**N = 45**	**N = 22**	**N = 29**	**N = 109**	**N = 391**
Anti-malarials						
Any first-line ACT	90.0 (82.5, 94.5)	85.6 (74.3, 92.4)	47.9 (27.8, 68.6)	2.8 (0.5, 15.6)	62.8 (51.7, 72.7)	70.9 (63.1, 77.6)
DHA PPQ	90.0 (82.5, 94.5)	85.6 (74.3, 92.4)	47.9 (27.8, 68.6)	2.8 (0.5, 15.6)	62.8 (51.7, 72.7)	70.9 (63.1, 77.6)
ASMQ FDC	0.0 (–)	0.0 (–)	0.0 (–)	0.0 (–)	0.0 (–)	0.0 (–)
Primaquine	0.0 (–)	0.0 (–)	0.0 (–)	0.0 (–)	0.0 (–)	0.0 (–)
Chloroquine	4.6 (2.0, 10.3)	7.7 (2.9, 19.1)	20.5 (9.7, 38.1)	67.1 (27.6, 91.6)	30.7 (20.1, 43.7)	19.7 (14.1, 26.9)
Other non-artemisinin therapy	1.3 (0.3, 5.4)	0.0 (–)	6.8 (1.9, 21.4)	0.0 (–)	1.8 (0.6, 5.2)	1.5 (0.7, 3.3)
Oral AMT	0.0 (–)	0.0 (–)	0.0 (–)	2.3 (0.4, 12.7)	0.0 (–)	0.2 (0.0, 1.2)
IV/IM artesunate	0.0 (–)	0.0 (–)	0.0 (–)	0.0 (–)	0.7 (0.1, 4.0)	0.2 (0.0, 1.3)
Availability of first-line ACT and malaria blood testing	80.4 (72.6, 86.3)	67.1 (52.5, 79.0)	23.6 (10.0, 46.1)	0.0 (–)	50.8 (40.2, 61.3)	59.4 (51.9, 66.6)
Availability of first-line ACT, blood testing not available	9.6 (6.1, 15.0)	18.5 (10.5, 30.5)	24.3 (9.4, 49.9)	2.8 (0.5, 15.6)	12.0 (7.0, 19.9)	11.5 (8.4, 15.4)

Approximately one-third of screened private for-profit health facilities (31.0%), one-fifth of pharmacies (20.5%), and 15.1% of itinerant drug vendors were stocking any anti-malarial on the day of the survey. Of 23,840 general retailers screened, only 0.2% were found to be stocking any anti-malarial.

Among anti-malarial-stocking private sector outlets, nearly two-thirds had malaria blood testing available (64.7%); 63.8% were stocking a malaria RDT, while 8.7% had malaria microscopy available. Availability of malaria blood testing was highest in private for-profit health facilities (83.0%) and pharmacies (70.1%). Over half of itinerant drug vendors were stocking a diagnostic test (60.4%).

Among those private sector outlets stocking any anti-malarial, 70.9% were stocking a first-line ACT, all of which was DHA PPQ rather than ASMQ FDC. Private for-profit health facilities and pharmacies had the highest availability of a first-line ACT (90.0 and 85.6%, respectively). Availability of a first-line ACT was also moderately high among anti-malarial-stocking itinerant drug vendors (62.8%). Fewer than half of anti-malarial-stocking drug stores (47.9%) and only 2.8% of anti-malarial-stocking general retailers had a first-line ACT available. Chloroquine was most commonly available among drug stores (20.5%), general retailers (67.1%), and itinerant drug vendors (30.7%). Only one package of oral AMT was found, and this product was audited at a general retail outlet.

### Market share

**Fig. 1:**
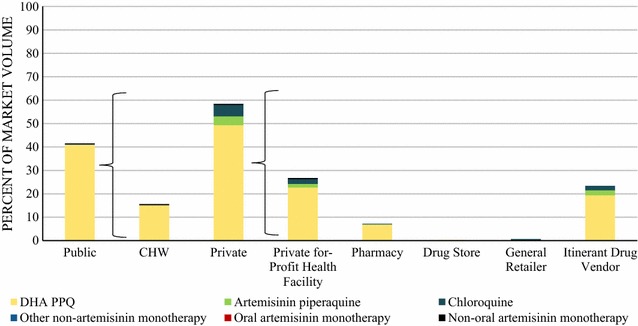
Anti-malarial market share

Figure 1 shows a detailed breakdown of the anti-malarial market share across sectors, outlet types, and anti-malarial type. Most of the anti-malarials were sold or distributed through the private sector (58.4%). Across the private sector, most of the anti-malarial market share was composed of private for-profit health facilities (26.7%) and itinerant drug vendors (23.4%).

In terms of the types of anti-malarials being sold or distributed, DHA PPQ contributed to the majority of the market share in both the public and private sectors (90.3% of the national market share). The private sector anti-malarial market share was also composed of chloroquine (4.9%), which was primarily distributed through private for-profit health facilities, general retailers, and itinerant drug vendors.

**Fig. 2:**
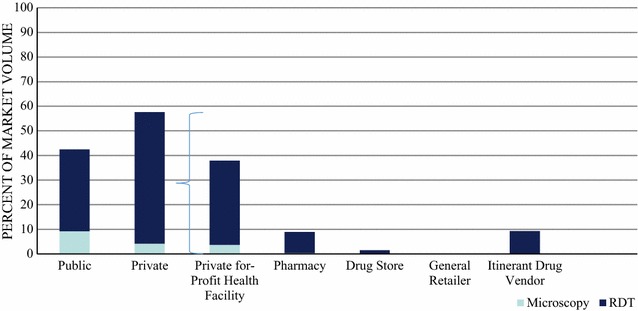
Malaria confirmatory testing market share

Figure 2 shows a detailed breakdown of the diagnostic market share across sectors, outlet types, and diagnostic test type. The majority of the diagnostic market share was composed of the private sector (57.6%), with most diagnostic tests provided through private for-profit health facilities (37.9%), itinerant drug vendors (9.1%), and pharmacies (8.6%). Most diagnostic testing was performed using RDT rather than microscopy.

### Private sector support

Table 5 shows a detailed breakdown of support reportedly received across private sector outlet types. Nearly half of all interviewed outlets (44.4%) reported having received any type of support. Nearly two-thirds of private for-profit health facilities (60.2%) and nearly three-fourths of pharmacies (71.7%) reported having received any type of support, while one in four itinerant drug vendors (25.5%) reported receiving any type of support.

**Table 5:** Percent of private sector outlets who reportedly received government or non-governmental support, and among outlets who received a support strategy, type of support received

	Private for-profit health facility	Pharmacy	Drug store	General retailer	Itinerant drug vendor	All private sector
	% (95% CI)	% (95% CI)	% (95% CI)	% (95% CI)	% (95% CI)	% (95% CI)
	**N = 326**	**N = 99**	**N = 46**	**N = 39**	**N = 235**	**N = 745**
Received any type of support	60.2 (53.6, 66.4)	71.7 (61.7, 80.0)	45.5 (29.7, 62.2)	3.2 (0.7, 13.8)	25.5 (18.8, 33.6)	44.4 (39.0, 49.9)
Among outlets with support	**N = 196**	**N = 69**	**N = 19**	**N = 1**	**N = 64**	**N = 349**
Report access to subsidized anti-malarials	58.3 (49.2, 66.9)	69.4 (58.7, 78.5)	26.8 (13.3, 46.6)	0.0 (–)	55.7 (44.4, 66.5)	57.5 (51.1, 63.7)
Report access to subsidized RDT	63.5 (54.0, 72.1)	69.1 (58.9, 77.7)	60.2 (36.4, 80.0)	0.0 (–)	58.6 (45.5, 70.7)	63.0 (55.9, 69.6)
Report received training in the past year on malaria diagnosis (RDT and/or microscopy) and/or the National treatment guidelines for malaria	65.3 (57.1, 72.7)	66.0 (53.2, 76.8)	36.1 (18.0, 59.2)	100.0 (–)	42.6 (31.5, 54.4)	59.1 (53.9, 64.2)
Report receiving a supervisory or regulatory visit within the past year	27.6 (21.7, 34.3)	17.8 (8.9, 32.6)	20.7 (5.5, 54.0)	0.0 (–)	8.6 (3.9, 18.0)	21.4 (16.7, 27.0)
Report keeping and reporting malaria caseload data to government or non-government organization	50.3 (40.0, 60.6)	22.2 (11.8, 37.9)	18.0 (4.0, 53.4)	0.0 (–)	21.1 (11.7, 35.0)	36.9 (27.8, 47.1)

Among outlets that reported receiving support, the most common types of support reportedly received were access to subsidized RDT (63.0%), training in the past year on malaria diagnosis or the national treatment guidelines for malaria (59.1%), and access to subsidized anti-malarials (57.5%). Only 21.4% reported receiving a supervisory or regulatory visit within the past year, and 36.9% reported keeping and reporting malaria caseload data to either the government or a non-governmental organization.

**Fig. 3:**
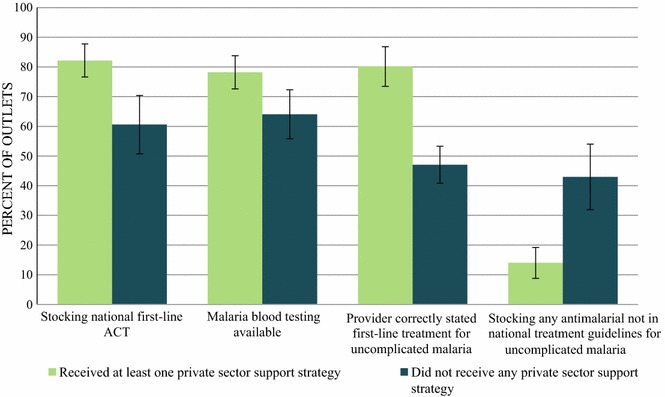
Key indicators by private sector support status

Figure 3 shows several key indicators showing anti-malarial and diagnostic market availability, and provider knowledge, according to outlet support status (the presence or absence of private sector support), while controlling for outlet type. Among private sector outlets stocking any anti-malarial, the proportion of outlets with a first-line ACT was higher among outlets that had reportedly received any type of support or engagement (hereinafter referred to as ‘supported’ outlets) compared to outlets that did not report receiving any support (82.1 and 60.6%, respectively). Similar results were observed for availability of malaria blood testing, where 78.2% of supported outlets had a malaria blood test available compared to 64.0% of unsupported outlets. With regards to provider knowledge, 80.2% of providers in supported outlets correctly stated the first-line treatment for uncomplicated malaria (either DHA PPQ or ASMQ FDC) compared to just 47.0% of providers in unsupported outlets. Finally, supported outlets were less likely to be stocking an anti-malarial not indicated in the national treatment guidelines for malaria compared to unsupported outlets (14.0% compared to 43.0%, respectively).

**Fig. 4:**
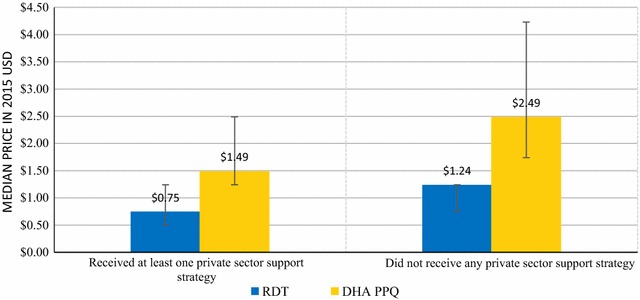
Median consumer prices for adult RDT and DHA PPQ, by private sector support status

Figure 4 shows median prices for malaria RDT and DHA PPQ according to outlet support. Outlets that reported having received any type of support reported a median consumer price of $0.75 for a malaria RDT compared to a median price of $1.49 in unsupported outlets. Similarly, the median price of one AETD of DHA PPQ in supported outlets was $1.24 compared to $2.49 in unsupported outlets.

## Discussion

The 2015 outlet survey findings provide contemporary evidence on the availability and market share of malaria commodities. Several positive outlet survey findings regarding the readiness and performance of the public and private sectors are observed: first-line ACT availability and distribution was widespread, and malaria diagnosis was commonplace—particularly in the public sector. However, the data also highlight key gaps across both sectors that must be addressed and which are discussed further in this section.

### Public sector readiness to test and treat for malaria

Readiness to appropriately manage malaria cases, measured through availability of malaria blood testing and a first-line ACT, was observed at only approximately two-thirds of public health facilities, indicating that around one in three public health facilities were lacking the capacity to both diagnose and appropriately treat uncomplicated malaria. According to Cambodia’s national treatment guidelines for malaria, and in order to achieve the universal coverage strategy described in the MEAF, all of the public sector outlet types must be equipped to test for and treat uncomplicated malaria. Moving forward, it will be critical to maintain a constant supply of malaria commodities. At the time of this survey, the CNM was developing the Logistic Management Information System (LMIS) to measure quantification, long-lasting insecticide-treated net (LLIN) demand, forecasting, and stock, with the aim of providing more regular reports and thus reducing the frequency of stock outs in the public sector [14]. The LMIS is expected to ideally move the country towards universal coverage by ensuring a constant supply of malaria commodities at all public sector outlets. Indeed, findings from other countries have supported this prediction, demonstrating that investments to strengthen management information systems can lend a more streamlined, demand-driven, and accountable procurement and supply chain system [15].

### The role of the private sector

The majority of private sector outlets screened were not in the business of stocking malaria commodities, with fewer than one in three private for-profit health facilities and one in five pharmacies stocking an anti-malarial. This reflects an overall decline in anti-malarial availability among these private sector outlets in recent years [5]. In 2015, malaria case management services in the private sector were concentrated among private for-profit health facilities and pharmacies, which are the only private sector outlet types authorized to distribute anti-malarial medicines [1]. The concentration of malaria commodities among authorized outlet types may reflect increased regulation of the private sector and/or may also be a result of a decline in provider incentives to stock anti-malarials, given declining burden and perhaps less consumer demand for malaria treatment. However, the relatively low availability of malaria commodities in the private sector indicates that a febrile patient seeking care in the private sector may have to approach multiple facilities to find one with malaria testing and treatment. This points to the importance of a referral system, such as the Private Sector SMS Referral System piloted in 2012 by the CNM and partners [16], as well as a need to scale up health services at the community level.

While most private sector outlets were not in the business of malaria case management, results nonetheless show that the private sector was responsible for the majority of anti-malarial distribution and malaria testing provision. This finding is consistent with other research in Cambodia and neighboring countries such as Lao PDR and Myanmar [17, 18]. Market share data also illustrate that, while a large portion of the private sector case management was channeled through private for-profit health facilities and pharmacies, there was also a significant contribution from itinerant drug vendors—an illicit and unregulated outlet type in Cambodia. Although itinerant drug vendors are not a formal or regulated outlet type, provider demographic results show that approximately one in five itinerant drug vendors reported having completed university or college, and nearly two in three itinerant drug vendors reported having a health qualification, mostly as a nurse or nursing officer. This suggests that these providers could be considered assets in improving appropriate malaria case management coverage, if they can be appropriately regulated and supervised.

The MEAF has outlined plans to target certain unlicensed providers and encourage them to obtain a licence in order to join the PPM programme, and it also describes goals to enforce existing laws that would prohibit operation of itinerant drug vendors. However, there may be some benefit from further exploration into whether itinerant drug vendors could be effectively licensed and regulated under the PPM programme. Several studies have documented success with the VMW programme [5, 19], which has increased access to appropriate malaria case management in many rural areas of Cambodia utilizing people with overall less education and fewer baseline health qualifications compared to itinerant drug vendors (Additional file 1). In sub-Saharan Africa, several malaria-endemic countries have documented improvements in provider knowledge and performance after implementation of strategies such as training and capacity-building, demand generation, quality assurance, and creating an enabling environment, all of which targeted the informal private sector, including itinerant drug vendors [20]. Furthermore, the WHO has recommended the engagement of itinerant drug vendors, where appropriate, as a method of improving home-based management of malaria [21, 22], and analyses by the U.S. Agency for International Development (USAID) have also concluded that policies through which national programmes engage with private providers—both formal and informal—can be beneficial to improving provision of care for malaria and other important health issues in developing countries [23]. Extending permission to test for and treat malaria to trained and supervised itinerant drug vendors may be an important strategy in Cambodia to accelerate universal coverage of confirmatory testing and appropriate malaria treatment and to continue expanding coverage to remote, rural populations.

### Private sector readiness and performance

Where anti-malarials were available in the private sector, the majority of anti-malarial stocking outlets had a first-line ACT available. However, nearly one in three private sector outlets were not stocking a first-line ACT and were primarily stocking chloroquine, which is no longer indicated for use in the national treatment guidelines for malaria. While the majority of anti-malarial-stocking private sector outlets had malaria diagnostic testing available, approximately one in three did not have testing available. These gaps in private sector readiness are a threat to appropriate management of suspected malaria cases, as they demonstrate a potential for presumptive anti-malarial treatment and/or treatment with medicines that are not indicated in the national treatment guidelines for malaria.

Most anti-malarials distributed by the private sector were DHA PPQ, a first-line ACT. However, chloroquine and artemisinin–piperaquine were also distributed in the private sector, indicating that some private sector providers were not in full alignment with the national treatment guidelines for malaria. Indeed, availability data show that all private sector outlet types were found to be stocking chloroquine, with the highest availability observed among general retailers. While availability and market share of a first-line treatment were high among private for-profit health facilities and pharmacies, chloroquine stubbornly persists and most notably among itinerant drug vendors, pointing to the need to completely remove this anti-malarial from the market. One strategy by which to achieve this goal would be to ban the import and sale of this anti-malarial, especially given evidence that a similar ban on oral artemisinin monotherapy in 2008 was found to be a successful measure to remove this anti-malarial from the market [5]. This is also addressed in the MEAF strategy, which stipulates that there will be increased efforts to ban the import and sale of anti-malarial drugs not in national treatment guidelines for malaria.

### Private sector support

Results from this study showed that access to any supportive intervention—including subsidized anti-malarials or RDT, training, supervisory or regulatory visits, or reporting caseload data—was associated with higher availability of a first-line ACT, higher availability of malaria testing, higher provider knowledge of first-line treatment guidelines for malaria, and lower availability of an anti-malarial not in the national treatment guidelines for malaria. In addition, the study showed that private sector outlets with access to any supportive intervention priced their malaria commodities lower than outlets that did not have access to any supportive intervention, meaning more affordable access to malaria testing and treatment for consumers.

These results suggest that strategies such as subsidies, training, and supervision can improve private sector readiness and performance, as has been demonstrated in other contexts [24]. However, this study was not designed to evaluate specific types of private sector support, nor was it able to compare the performance of PPM outlets with non-PPM outlets. There is further need to examine the outcomes associated with the various aspects of the PPM programme to identify where there may be a need to strengthen specific components. The extent to which the measures of access to supportive interventions, as outlined in this paper, can be used to inform specific strategies relating to the PPM programme is limited.

Results from this study reflect positively on the work that has been done to-date in Cambodia to engage the private sector. However, there is a need to increase coverage of private sector support and, in doing so, more evidence about the performance of current strategies is needed in order to facilitate efficient and effective progress in the private sector. As intended under the PPM programme, current strategies appear to be reaching private health facilities and pharmacies to a much greater extent than itinerant drug vendors. As noted above, extending private sector support to itinerant drug vendors may be an important strategy for improving overall private sector readiness and performance. In addition, investigation into the merits of other types of private sector strategies, such as integration of RDT financial incentives and information, education, and counseling, may be useful interventions as evidenced among the informal private sector in neighboring Myanmar [25]. Finally, future research that specifically addresses the PPM programme may be merited in order to better understand how this strategy has affected anti-malarial and diagnostic market performance to date and in order to more specifically inform policy decisions relating to this programme.

### Gaps in availability of ASMQ FDC and primaquine

In its continuing effort to keep one step ahead of drug resistance, Cambodia’s national treatment guidelines for malaria changed in 2014 in response to emerging resistance to DHA PPQ, such that ASMQ FDC is now recommended in geographic areas with DHQ PPQ failure. As of June 2015, failure rates of DHA PPQ have reached over 60% in certain areas of the country, pointing to the immediate need to ensure access to ASMQ FDC [1]. During data collection for this survey, which took place in August and September of 2015, ASMQ FDC was not found. The absence of this anti-malarial in the market may be due in part to a variety of challenges with manufacturing and procurement. Cambodia has faced procurement challenges in the past, most notably after the switch in malaria treatment guidelines to DHA PPQ in 2010, when a lack of suitable manufacturers led to a significant delay and subsequent stock out of first-line anti-malarials in both the public and private sectors [5]. In light of the constantly changing epidemiology of malaria in Cambodia and the Greater Mekong Subregion (GMS) as a whole, it is important that the country is able to respond quickly to changing treatment recommendations and thus avoid lags in the stocking of appropriate treatments. One option to consider are parallel procurement systems whereby two or more types of anti-malarial are stocked in-country to guarantee the availability of an appropriate anti-malarial depending on the drug resistance, while accepting that drug wastage will be an inevitable reality in the drive toward elimination [5]. Other considerations may include investment into a centralized procurement system, with trained personnel, storage capacity, infrastructure and IT enablement in order to forecast stock and supply, and distribution of sufficient quantities of anti-malarials with minimal delays [26]. In addition, any identification of treatment failure should signal the need to start immediately forecasting sufficient stock for new first-line treatments.

The MEAF and the national treatment guidelines for malaria stipulate the use of primaquine to prevent *P. falciparum* transmission and *P. vivax* relapse, indicating that it should be provided along with a first-line ACT for both types of malaria. This study found that primaquine was universally unavailable at the time of the survey. This may reflect hesitancy to operationalize the use of primaquine without a feasible way to test first for G6PD deficiency [27]. However, WHO recommendations and supporting publications demonstrate that a low dose of primaquine can be safely administered regardless of G6PD status [28–30]. As Cambodia scales up access to primaquine, approaches used in neighboring countries such as Myanmar, Thailand, and Vietnam may be useful to consider, as governments have limited the use of primaquine to facilities that are equipped to either test and/or monitor for signs of G6PD deficiency. However, it should be noted that outlet survey evidence from Myanmar and Thailand illustrates that primaquine availability in these facilities was generally lower compared to other first-line treatments for uncomplicated malaria [31]. This suggests that there may be issues in maintaining constant supply or may demonstrate concerns by the governments that facilities are not adequately equipped to either test and/or monitor for signs of G6PD deficiency. Building on evidence from other countries that have historically included and implemented the use of primaquine in their treatment policy will be helpful to facilitate full implementation of the national treatment guidelines for malaria in Cambodia.

### Limitations

Despite its many strengths, the ACTwatch outlet survey has several limitations which have been described in detail elsewhere [8, 32]. Notably, the survey had a cross sectional design, which limits the conclusions that can be drawn about causality as it relates to access to supportive interventions and performance indicators in the private sector. It is also acknowledged that, due to increased regulation of the private sector, especially as it relates to the stocking of oral artemisinin monotherapy, providers may have a disincentive to accurately report certain information, such as the stocking of artemisinin monotherapy or the stocking of any malaria commodities in outlets which were unlicensed.

## Conclusions

As Cambodia steps into an era of malaria elimination, evidence on the availability and distribution of first-line treatment for malaria and malaria diagnostic testing in the public and private sectors is critical. Evidence from the last ACTwatch outlet survey implemented in 2015 illustrates that there is a strong foundation for meeting national malaria elimination goals: first-line ACT availability and distribution was widespread, and malaria diagnosis was commonplace—particularly in the public sector. This evidence can serve as a benchmark for guiding the implementation of strategies outlined in the MEAF as well as other regional initiatives to address elimination activities. The private sector remains responsible for the majority of malaria testing and treatment in Cambodia, indicating that strategies to effectively support the private sector are critical to continued progress. Identifying other regulatory strategies or supportive interventions to address anti-malarial availability and distribution by unauthorized itinerant drug vendors is needed.

## Additional file

**Additional file 1.** Demographic information of private sector providers, by outlet type.

## Abbreviations

ACT: artemisinin-based combination therapy
AETD: adult equivalent treatment dose
ASMQ: artesunate–mefloquine
CHW: community health worker
CNM: Cambodia’s National Centre for Parasitology, Entomology and Malaria Control
DEF: Department of Food and Drug
DHA PPQ: dihydroartemisinin-piperaquine
FDC: fixed-dose combination
GMS: Greater Mekong Sub-region
G6PD: glucose-6-phosphate dehydrogenase
IV: intravenous
IM: intramuscular
LLIN: long-lasting insecticide-treated net
LMIS: logistic management information system
MEAF: Malaria Elimination Action Framework
MOH: Ministry of Health
OD: operational districts
RDT: rapid diagnostic test
PPM: public private sector mix
PPS: probability proportional to size
PSI: Population Services International
USAID: U. S. Agency for International Development
VMW: village malaria worker
WHO: World Health Organization
