# Supplementary material for: Evidence on anti-malarial and diagnostic markets in Cambodia to guide malaria elimination strategies and policies
Source: Malar J. 2017 Apr 25;16:171. doi: 10.1186/s12936-017-1807-y (PMC5404333; doi:10.1186/s12936-017-1807-y)
Supplement: Supplementary file 1 — Additional file 1. Demographic information of private sector providers, by outlet type. [file 12936_2017_1807_MOESM1_ESM.docx]

**Additional file 1: Demographic information of private sector providers, by outlet type**

|  | **Public Health Facility** | **Community Health Worker** | **ALL Public Sector** | **Private**  **for-Profit**  **Health Facility** | **Pharmacy** | **Drug Store** | **General Retailer** | **Itinerant**  **Drug Vendor** | **ALL Private Sector** |
| --- | --- | --- | --- | --- | --- | --- | --- | --- | --- |
|  | **N=142** | **N=415** | **N=557** | **N=319** | **N=99** | **N=46** | **N=39** | **N=235** | **N=738** |
| **Among providers at eligible and interviewed outlets, median:** | **Median**  **[IQR]** | **Median**  **[IQR]** | **Median**  **[IQR]** | **Median**  **[IQR]** | **Median**  **[IQR]** | **Median**  **[IQR]** | **Median**  **[IQR]** | **Median**  **[IQR]** | **Median**  **[IQR]** |
| Age | 42 | 40 | 41 | 41 | 38 | 49 | 42 | 47 | 44 |
|  | [30, 47] | [29, 52] | [29, 51] | [31, 49] | [30, 46] | [29, 53] | [33, 55] | [35, 55] | [32, 52] |
| Number of years worked at outlet | 12 | 3 | 4 | 5 | 8 | 4 | 8 | 10 | 7 |
|  | [3, 17] | [2, 6] | [2, 8] | [2, 13] | [2, 16] | [2, 18] | [3, 20] | [4, 20] | [3, 15] |
| **Among senior providers at eligible and interviewed outlets, percentage:** | **%**  **(95% CI)** | **%**  **(95% CI)** | **%**  **(95% CI)** | **%**  **(95% CI)** | **%**  **(95% CI)** | **%**  **(95% CI)** | **%**  **(95% CI)** | **%**  **(95% CI)** | **%**  **(95% CI)** |
| Male | 69.0 | 48.7 | 53.3 | 71.6 | 47.4 | 49.6 | 14.1 | 77.7 | 65.4 |
|  | (59.1, 77.3) | (43.5, 54.0) | (48.6, 57.9) | (65.5, 76.9) | (38.9, 56.1) | (35.2, 64.1) | (7.9, 23.9) | (71.8, 82.6) | (60.6, 69.8) |
| **Among senior providers at eligible and interviewed outlets, highest level of education completed:** | **%**  **(95% CI)** | **%**  **(95% CI)** | **%**  **(95% CI)** | **%**  **(95% CI)** | **%**  **(95% CI)** | **%**  **(95% CI)** | **%**  **(95% CI)** | **%**  **(95% CI)** | **%**  **(95% CI)** |
| No formal education | 0.7 | 4.8 | 3.9 | 0.0 | 0.0 | 1.7 | 14.1 | 2.3 | 1.9 |
|  | (0.1, 4.3) | (2.9, 8.0) | (2.4, 6.4) | - | - | (0.3, 9.7) | (6.5, 27.9) | (0.7, 6.8) | (0.9, 3.9) |
| Some primary school | 1.7 | 40.7 | 31.9 | 1.8 | 0.8 | 21.8 | 49.9 | 14.8 | 11.0 |
|  | (0.5, 5.1) | (35.8, 45.7) | (27.6, 36.5) | (0.8, 4.0) | (0.1, 5.1) | (10.4, 40.0) | (37.9, 61.8) | (9.3, 22.7) | (7.9, 15.2) |
| Completed primary school | 4.6 | 13.1 | 11.2 | 2.3 | 1.4 | 8.5 | 0.0 | 6.9 | 4.0 |
|  | (2.0, 10.5) | (9.9, 17.3) | (8.6, 14.5) | (1.0, 5.2) | (0.4, 5.3) | (3.7, 18.7) | - | (3.9, 11.8) | (2.7, 6.1) |
| Some secondary school | 15.5 | 35.8 | 31.2 | 10.3 | 24.5 | 25.1 | 31.5 | 32.1 | 22.1 |
|  | (10.6, 22.2) | (31.7, 40.1) | (27.8, 34.9) | (7.4, 14.1) | (16.7, 34.6) | (14.9, 39.0) | (18.5, 48.3) | (27.0, 37.8) | (19.1, 25.4) |
| Completed secondary school | 23.4 | 4.7 | 8.9 | 15.3 | 24.5 | 24.1 | 4.5 | 15.8 | 16.3 |
|  | (16.6, 31.9) | (3.0, 7.3) | (6.7, 11.7) | (11.1, 20.6) | (16.2, 35.3) | (12.8, 40.7) | (0.9, 18.7) | (11.8, 20.7) | (13.7, 19.2) |
| Some university/college | 9.4 | 0.3 | 2.4 | 12.5 | 8.3 | 12.4 | 0.0 | 6.9 | 9.1 |
|  | (4.8, 17.6) | (0.1, 1.9) | (1.3, 4.4) | (8.1, 18.9) | (4.7, 14.2) | (6.9, 21.3) | - | (4.2, 11.1) | (6.8, 12.1) |
| Completed university/college | 44.6 | 0.6 | 10.5 | 57.9 | 40.4 | 6.4 | 0.0 | 21.3 | 35.5 |
|  | (35.0, 54.7) | (0.2, 1.8) | (7.5, 14.4) | (50.5, 64.9) | (29.6, 52.3) | (2.4, 15.9) | - | (15.5, 28.5) | (30.6, 40.8) |
| **Among eligible and interviewed outlets, proportion with at least one provider who had:** | **%**  **(95% CI)** | **%**  **(95% CI)** | **%**  **(95% CI)** | **%**  **(95% CI)** | **%**  **(95% CI)** | **%**  **(95% CI)** | **%**  **(95% CI)** | **%**  **(95% CI)** | **%**  **(95% CI)** |
| Received training on malaria national treatment guidelines for malaria in the previous 12 months | 35.7  (28.0, 44.3) | 66.5  (58.7, 73.5) | 59.6  (52.9, 66.0) | 36.6  (29.3, 44.6) | 43.3  (31.9, 55.4) | 16.4  (7.8, 31.2) | 3.2  (0.7, 13.8) | 9.3  (6.3, 13.7) | 23.9  (19.5, 28.9) |
| Received training on malaria diagnosis in the previous 12 months | 33.3 | 59.4 | 53.5 | 34.8 | 41.5 | 15.2 | 3.2 | 7.3 | 22.2 |
|  | (24.5, 43.5) | (51.7, 66.7) | (46.6, 60.3) | (27.1, 43.3) | (31.0, 52.7) | (6.8, 30.5) | (0.7, 13.8) | (4.7, 11.3) | (17.8, 27.4) |
| Any health-related qualification | 100.0 | 88.8 | 91.3 | 98.3 | 78.6 | 64.4 | 14.2 | 63.2 | 75.5 |
|  | - | (78.5, 94.5) | (83.4, 95.6) | (96.3, 99.2) | (64.8, 88.0) | (48.1, 77.9) | (8.0, 24.0) | (55.2, 70.5) | (70.7, 79.7) |
| Pharmacist | 11.4 | 0.0 | 2.6 | 4.1 | 41.2 | 4.7 | 0.0 | 2.6 | 7.6 |
|  | (7.5, 17.0) | - | (1.6, 4.2) | (2.4, 7.1) | (30.8, 52.4) | (0.9, 20.7) | - | (1.2, 5.5) | (5.6, 10.1) |
| Medical doctor | 16.3 | 0.0 | 3.7 | 32.3 | 15.1 | 0.0 | 0.0 | 5.1 | 16.5 |
|  | (11.4, 22.8) | - | (2.4, 5.6) | (24.2, 41.7) | (9.7, 22.7) | - | - | (2.4, 10.5) | (12.5, 21.4) |
| Nurse / Nursing officer | 96.2 | 1.1 | 22.5 | 72.3 | 40.7 | 34.8 | 0.0 | 43.1 | 50.8 |
|  | (89.7, 98.7) | (0.4, 3.0) | (18.3, 27.3) | (65.3, 78.4) | (30.9, 51.3) | (21.6, 50.8) | - | (34.2, 52.4) | (45.8, 55.8) |
| Midwife | 90.0 | 0.4 | 20.5 | 27.4 | 13.7 | 22.9 | 0.0 | 10.5 | 17.6 |
|  | (78.3, 95.7) | (0.1, 2.2) | (16.4, 25.4) | (22.8, 32.5) | (8.8, 20.8) | (12.8, 37.6) | - | (6.9, 15.6) | (14.9, 20.7) |
| Laboratory technician / Laboratory assistant | 21.0 | 1.0 | 5.4 | 14.4 | 6.8 | 6.6 | 5.6 | 6.3 | 9.6 |
|  | (14.8, 28.9) | (0.2, 3.9) | (3.7, 8.0) | (10.0, 20.4) | (3.8, 12.0) | (1.5, 25.1) | (1.7, 16.6) | (3.5, 11.1) | (7.2, 12.6) |
| Pharmacy technician / Pharmacy assistant | 12.7 | 0.0 | 2.9 | 2.7 | 7.8 | 5.7 | 0.0 | 4.3 | 3.8 |
|  | (8.1, 19.4) | - | (1.7, 4.7) | (1.5, 4.8) | (3.7, 15.6) | (1.5, 19.4) | - | (2.3, 8.0) | (2.5, 5.8) |
| Health assistant / Nursing assistant / Nursing aid | 34.4  (26.2, 43.7) | 0.1  (0.0, 0.6) | 7.8  (5.6, 10.8) | 9.4  (6.0, 14.4) | 7.8  (3.9, 15.2) | 18.2  (8.8, 33.7) | 3.5  (0.5, 19.7) | 5.3  (3.2, 8.7) | 7.9  (6.0, 10.3) |
| Village Malaria Worker / Mobile Malaria Worker / Plantation Malaria Worker | 3.0  (1.4, 6.3) | 87.9  (77.9, 93.7) | 68.9  (60.5, 76.3) | 2.2  (0.9, 5.3) | 0.0  - | 6.6  (1.9, 20.7) | 5.1  (1.8, 14.1) | 1.5  (0.5, 4.2) | 2.2  (1.2, 3.9) |
